# Association of Preoperative Plasma D-Dimer and Fibrinogen and Osteosarcoma Outcome

**DOI:** 10.3389/fonc.2022.699295

**Published:** 2022-04-08

**Authors:** Yanchuan Pu, Jin Wang, Jianshu Wang, Shizhong Wang

**Affiliations:** ^1^ Department of Orthopedics, Wuwei City People’s Hospital, Wuwei, China; ^2^ Department of Bone Oncology, Gansu Cancer Hospital, Lanzhou, China

**Keywords:** progression-free survival, osteosarcoma, prognosis, overall survival, d-dimer, plasma fibrinogen

## Abstract

**Objective:**

We aimed to evaluate the utility of preoperative D-dimer and plasma fibrinogen (PF) levels as useful markers for predicting the clinical value of patients with osteosarcoma.

**Methods:**

145 enrolled patients with osteosarcoma were studied retrospectively. We determined the critical values of D-dimer and PF by receiver operating characteristic curve analysis. Cox regression analysis was used to assess prognostic role of the D-dimer and PF levels among osteosarcoma patients.

**Results:**

The critical values of D-dimer and PF were calculated to be 0.46 µg/mL and 3.34 mg/mL, respectively. Upregulation of D-dimer and PF showed positive correlations with a higher clinical stage, tumour metastasis and recurrence. Survival curve results confirmed that osteosarcoma patients with higher levels of D-dimer and PF predicted worse overall survival (OS) and progression-free survival (PFS). Moreover, only a high D-dimer level was associated with a shorter OS (P = 0.013) and PFS (P = 0.042) in both the univariate and multivariate analysis.

**Conclusion:**

Elevated preoperative D-dimer levels are correlated with aggressive clinicopathological features and poor survival outcomes, which indicates that assessment of the D-dimer could be a useful prognostic marker in osteosarcoma.

## Introduction

Osteosarcoma is the most common primary bone tumour in young patients. The peak incidence of osteosarcoma occurs in patients who are 18 years old (1). Though comprehensive treatments (neoadjuvant chemotherapy, surgery and adjuvant chemotherapy) have been developed, the 5-year overall survival (OS) rate of patients with osteosarcoma without metastasis is less than 70%, while that of patients with metastatic disease is less than 20% (2). Thus, new prognostic markers to identify metastatic disease in osteosarcoma are essential and could be helpful for conducting a systematic assessment of the prognosis of osteosarcoma and providing accurate guidance for individualized clinical treatment.

To date, several investigators have confirmed an association between the activation of the coagulation/fibrinolysis system and tumour progression (3, 4). Based on the available literature, the two parts (plasma fibrinogen (PF) and D-dimer) of the plasminogen-plasmin and coagulation system may be related to prognosis in osteosarcoma. Fibrinogen, a molecule synthesized by liver parenchyma cells in response to cytokine stimulation, participates in both the inflammatory response and carcinoma progression (5). Several studies have suggested that PF levels are upregulated in several carcinomas and might be associated with tumour progression and metastasis (6–8). However, no study has reported the relationship between PF and osteosarcoma prognosis.

Plasma D-dimer is a signal of hyperfibrinolysis and an end-product of fibrin degradation that can be detected in blood samples (9). An elevated D-dimer level before surgery has been shown to be closely related to the occurrence and development of a variety of malignancies, such as gastric cancer (10), ovarian cancer (11), and gynaecological carcinomas (12). Huang et al. (13) demonstrated that D-dimer is a biomarker for chemotherapy response in patients with osteosarcoma. In addition, other data also confirmed that D-dimer was a predictor of patients with metastatic osteosarcoma treated with second-line chemotherapy (14). However, its effectiveness in prognosis prediction of osteosarcoma has not been fully characterized.

Hence, we examined the D-dimer and PF levels in nonmetastatic and metastatic primary osteosarcoma samples to elucidate the relationship between these markers and the associated clinical features.

## Materials and Methods

### Patient Selection

Between July 2006 and December 2015, 160 consecutive pathologically confirmed osteosarcoma patients who were treated at Wuwei People’s Hospital (Wuwei, China) were enrolled. Patients were followed until January 30, 2021. The mean follow-up was 86 months (range 3–123). Only pathological samples taken before chemoradiotherapy were collected, and none of the patients received chemoradiotherapy before the surgery.

The inclusion criteria were as follows: (1) osteosarcoma confirmed by pathology; (2) patients undergoing surgery in our hospital from July 2006 to December 2015; and (3) sufficient clinical data in the medical records. Patients who received second-line chemotherapy. Two different pathologists assessed the histopathology of each sample to confirm the diagnosis of osteosarcoma according to the World Health Organization’s classification of bone tumours. Ultimately, 145 patients diagnosed with osteosarcoma were included, including 104 men and 41 women. The Ethics Committees of Wuwei City People’s Hospital approved the research.

Clinical features, including sex, age, tumour size, clinical stage, pathological fracture, metastasis and recurrence status, physical examination and smoking status, were noted. The levels of D-dimer and PF were detected in all patients within 7 days before the first treatment. PF and D-dimer levels were tested using an automatic coagulation analyser (Stago, France). PF and D-dimer levels were measured by the clotting method and immunoturbidimetry assays, respectively.

### Optimal Prognostic Cut-Off Values for D-Dimer and PF

**Figure 1:**
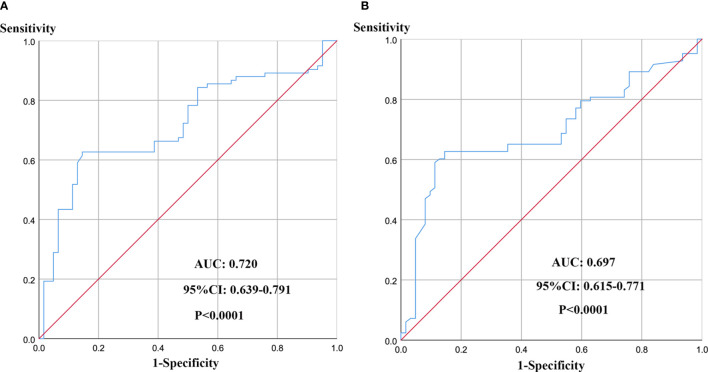
ROC curve analysis for determination of optimal cut-off values of D-dimer **(A)** and PF **(B)** levels in 5-year OS.

The mean D-dimer and PF levels were 0.56 µg/mL (SD, ± 0.18) and 3.44 g/L (SD, ± 1.96), respectively. According to the findings of the receiver operating characteristic curve (ROC) analysis, the optimum cut-off values were 0.46 µg/mL for preoperative D-dimer (**Figure 1A**) and 3.34 mg/mL for preoperative PF (**Figure 1B**). The maximum area under the curve (AUC) values for the ROC curves corresponding to D-dimer and PF were 0.720 (95% CI 0.639 to 0.791) and 0.697 (95% CI 0.615 to 0.771), respectively.

The sensitivities of D-dimer and PF were 0.854 and 0.855, respectively, while the specificities were 0.626 and 0.627, respectively. According to the optimal cut-off values for D-dimer and PF, the patients were divided into the following groups: high PF group (> 3.34 mg/mL; n = 84) and low PF group (≤ 3.34 mg/mL; n = 61) and high D-dimer group (> 0.46 µg/mL; n = 95) and low D-dimer group (≤ 0.46 µg/mL; n = 50).

### Statistical Methods

SPSS 26.0 (IBM, Armonk, NY, USA) and GraphPad Prism 8.0 (GraphPad Software, La Jolla, CA) software were used for statistical analysis. ROC curves for 5-year OS prediction were established to assess the optimal cut-off values of coagulation parameters so the parameters could be treated as binary variables. A chi-square test was performed to analyse the relationships between clinicopathological factors and coagulation indexes. Survival was assessed by creating a Kaplan-Meier curve; paired comparisons were made using a log-rank test. The univariate and multivariate analyses was used to estimate the correlation between D-dimer and PF and survival outcome (OS and progression-free survival (PFS). P < 0.05 was considered to indicate statistical significance.

## Results

### Clinicopathological Features

Between July 2006 and December 2015, 160 patients with osteosarcoma were first diagnosed in our hospital. After employing the strict inclusion and exclusion criteria, 145 osteosarcoma patients were included. The study cohort consisted of 104 (71.7%) men and 41 (28.3%) women among the 145 patients, with a median age of 18 years (8-53 years). There were 69 patients (47.6%) with tumour stage (I-II) and 76 patients (52.4%) with tumour stage (III-IV). The tumour diameter was 8 cm or less for 70 patients (48.3%) and more than 8 cm for 75 patients (51.7%). In addition, 73 patients (50.3%) experienced metastasis, including pulmonary metastases (46) and bone metastases (27), while the other 72 patients did not experience metastasis (49.7%). Seventy-six patients (52.4%) experience tumour recurrence, including 61 who experienced it once, 12 who experienced it twice, and 2 patients who experienced it three times, while the other 69 patients did not experience tumour recurrence (47.6%). Twenty-seven patients (18.6%) experienced pathological fracture during the follow-up.

### Associations of D-Dimer and PF Levels With Clinicopathological Features

As shown in **Table 1**, the baseline characteristics of osteosarcoma patients based on the D-dimer and PF groups are shown. Our results indicated that metastasis and recurrence at diagnosis were significantly correlated with a high D-dimer level (P = 0.012 and P = 0.030) and a high PF level (P < 0.001 and P = 0.019). Advanced tumour stage was closely associated with high D-dimer and PF levels (P = 0.012 and P = 0.044). The distribution of sex, age, tumour size, pathological fracture, and operation mode did not differ significantly between the D-dimer and PF groups.

**Table 1:** Associations of PF and D-dimer with clinicopathological factors of 145 patients with osteosarcoma.

Characteristic	Total (%)	PF			D-dimer		
		>3.34 g/L	≤3.34 g/L	*P*	>0.46 µg/mL	≤0.46 µg/mL	*P*
**Gender**							
Male	104 (71.7)	58	46	0.401	70	34	0.470
Female	41 (28.3)	26	15		25	16	
**Age, years**							
<18	64 (44.1)	37	27	0.979	42	22	0.981
≥18	81 (55.9)	47	34		53	28	
**Tumour size, cm**							
<8	75 (51.7)	40	35	0.246	47	28	0.455
≥8	70 (48.3)	44	26		48	22	
**Pathological fracture**							
No	118 (81.4)	68	50	0.877	77	41	0.889
Yes	27 (18.6)	18	13		18	9	
**Operation**							
Amputation	18 (12.4)	11	7	0.770	10	8	0.342
Salvage	127 (87.6)	73	54		85	42	
**Clinical stage**							
I/II	69 (47.6)	34	35	0.044	38	31	0.012
III/IV	76 (52.4)	50	26		57	19	
**Metastasis**							
Absent	72 (49.7)	30	42	<0.001	40	32	0.012
Present	73 (50.3)	54	19		55	18	
**Recurrence**							
Absent	69 (47.6)	33	36	0.019	39	30	0.030
Present	76 (52.4)	51	25		56	20	

### Prognostic Factors for OS in Patients With Osteosarcoma

**Figure 2:**
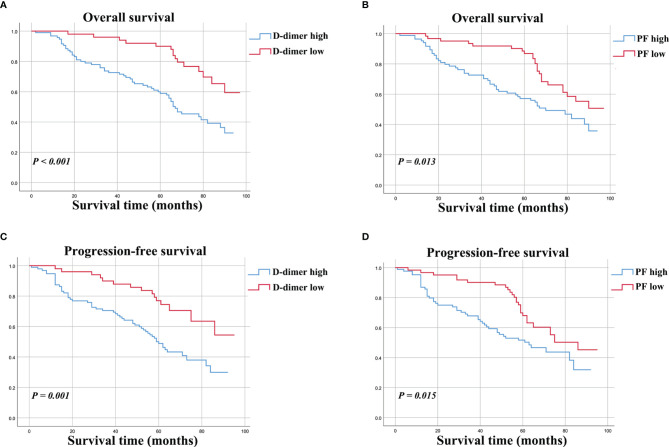
Kaplan-Meier curves for OS **(A, B)** and PFS **(C, D)** for osteosarcoma patients stratified by preoperative D-dimer and PF.

The potential prognostic value of D-dimer (P < 0.001) and PF (P = 0.013) levels for predicting OS was evaluated by Kaplan-Meier survival analysis (**Figures 2A, B** **)**. The D-dimer-high and D-dimer-low groups showed 5-year OS rates of to 52.4% and 76.6%, respectively. The PF-high and PF-low groups showed 5-year OS rates of to 53.7 and 77.1%, respectively.

The univariate analysis identified tumour clinical stage, metastasis, recurrence, PF level and D-dimer level (all P < 0.001). In addition, the multivariate Cox proportional hazard model suggested that several factors [metastasis (P < 0.001), recurrence (P = 0.006), and D-dimer level (P = 0.013)] were associated with the OS of patients (**Table 2**).

**Table 2:** Univariate and multivariate analyses of PF and D-dimer expression in relation to OS in patients with osteosarcoma.

Characteristic	Univariate			Multivariate		
	Hazard Ratio	95% CI	*P value*	Hazard Ratio	95% CI	*P value*
**Age, years**						
≥ 18 *vs* < 18	1.189	0.740-1.909	0.474	1.381	0.849-2.246	0.194
**Gender**						
Male *vs* Female	0.875	0.512-1.494	0.625	0.253	0.058-1.104	0.068
**Tumour size, cm**						
≥8 *vs <*8	1.504	0.937-2.412	0.091	0.898	0.513-1.571	0.706
**Pathological fracture**						
Present *vs* Absent	1.264	0.703-2.273	0.434	0.886	0.471-1.668	0.709
**Operation**						
Amputation *vs* Salvage	1.160	0.679-1.981	0.586	4.218	0.964-18.462	0.056
**Clinical stage**						
III/IV *vs* < I/II	2.428	1.482-3.977	<0.001	1.371	0.440-4.270	0.587
**Metastasis**						
Present *vs* Absent	3.119	1.843-5.276	<0.001	2.661	1.551-4.563	<0.001
**Recurrence**						
Present *vs* Absent	2.324	1.425-3.788	0.001	2.002	1.219-3.287	0.006
**PF**						
High *vs* Low	1.833	1.124-2.988	0.015	1.069	0.625-1.826	0.808
**D-dimer**						
High *vs* Low	2.782	1.571-4.928	<0.001	2.117	1.174-3.818	0.013

### Prognostic Factors for PFS in Patients With Osteosarcoma

The survival curve indicated that osteosarcoma patients with higher D-dimer (P = 0.001) and PF (P = 0.015) levels had poorer PFS (**Figures 2C, D** **)**.

The univariate analysis indicated that D-dimer (P = 0.002) and PF (P = 0.017) level were significantly related to PFS, as were clinical stage, metastasis, and recurrence (all P < 0.05). Furthermore, the multivariate analysis confirmed that elevated D-dimer levels (P = 0.042), metastasis (P = 0.001), and recurrence (P = 0.001) remained independent factors of PFS in osteosarcoma patients (**Table 3**).

**Table 3:** Univariate and multivariate analyses of PF and D-dimer expression in relation to PFS in patients with osteosarcoma.

Characteristic	Univariate			Multivariate		
	Hazard Ratio	95% CI	*P value*	Hazard Ratio	95% CI	*P value*
**Age, years**						
≥ 18 *vs* < 18	1.187	0.739-1.906	0.478	1.343	0.825-2.185	0.236
**Gender**						
Male *vs* Female	0.863	0.505-1.476	0.591	0.295	0.082-1.062	0.062
**Tumour size, cm**						
≥ 8 *vs* < 8	1.437	0.897-2.301	0.132	0.942	0.541-1.637	0.831
**Pathological fracture**						
Present *vs* Absent	1.375	0.763-2.480	0.289	1.002	0.526-1.907	0.996
**Operation**						
Amputation *vs* Salvage	1.177	0.689-2.011	0.743	3.534	0.980-12.748	0.054
**Clinical stage**						
III/IV *vs* < I/II	2.659	1.617-4.374	<0.001	1.342	0.422-4.267	0.619
**Metastasis**						
Present *vs* Absent	2.806	1.679-4.690	<0.001	2.404	1.419-4.074	0.001
**Recurrence**						
Present *vs* Absent	2.587	1.581-4.234	0.001	2.324	1.406-3.841	0.001
**PF**						
High *vs* Low	1.814	1.110-2.964	0.017	1.145	0.667-1.965	0.624
**D-dimer**						
High *vs* Low	2.511	1.418-4.444	0.002	1.848	1.022-3.343	0.042

## Discussion

Osteosarcoma is a highly complex and heterogeneous neoplasm with different recurrence and progression rates. Understanding its molecular pathogenesis and identifying specific biomarkers are necessary for improving the prognosis of osteosarcoma. It has been widely recognized that there is a link between tumours and haemostasis (15). The plasma D-dimer and PF levels have been widely considered reliable biomarkers of haemostasis and fibrinolysis activation. Previous research confirmed that high levels of these two coagulation parameters (D-dimer and PF) are risk factors predicting worse survival outcomes in various solid tumours. However, few studies have focused on the effect of the two coagulation parameters in patients with osteosarcoma. Therefore, we evaluated the prognostic significance of these two coagulation parameters in a cohort of osteosarcoma patients.

In the present research, our results showed the following: (a) upregulation of D-dimer and PF predicted a shorter survival time for osteosarcoma patients; (b) D-dimer and PF levels were closely related to a more advanced tumour stage, metastasis and recurrence in osteosarcoma patients; (c) aberrant D-dimer and PF levels were not strongly related to sex, age, tumour size, pathological fracture, or operation mode in osteosarcoma patients; and (d) only the D-dimer can be an independent prognostic parameter in osteosarcoma. This result may be because osteosarcoma patients are more likely to have high levels of D-dimer and PF. Although further validation and research are needed, our findings provide new insights into the prognostic significance of D-dimer and PF levels in osteosarcoma patients.

The biological mechanism of D-dimer explains its prognostic significance in osteosarcoma. In osteosarcoma, cancer cells can alter the balance among the coagulation, anticoagulation, and fibrinolytic systems by various mechanism, resulting in hyperactivity of coagulation and fibrinolysis system *via* a number of mechanisms, resulting in hyperactivity in the coagulation and fibrinolysis systems (16). Plasma D-dimer is a well-known stable degradation product of fibrin that increases during fibrin formation and enhanced fibrinolysis (17). Moreover, the associations between plasma D-dimer and circulating tumour cells (CTCs) observed in relevant studies suggests that the activation of the coagulation cascade could play a crucial role in promoting early tumour metastasis (18).

As an acute-phase glycoprotein, PF is usually related to the maintenance of haemostasis and plays a vital role in tumour cell viability, migration, and invasion (19). For instance, cell line model studies have shown that a high concentration of fibrinogen can induce epithelial-mesenchymal transition (EMT) by raising the expression of vimentin and decreasing the expression of E-cadherin, thus enhancing the invasion and migration of cancer cells (20). In addition, fibrinogen can bind to fibroblast growth factors and promote the binding of these growth factors with their receptors on the surface of cancer cells, further enhancing cancer growth and metastasis (21, 22).

Our study is the first to explore the relationships of the levels of D-dimer and PF with the clinicopathological characteristics and survival outcome in osteosarcoma. The concentrations of D-dimer and PF before surgery may also be useful markers for predicting metastasis in osteosarcoma patients following resection. D-dimer and PF can be easily detected in clinical laboratories, and they are effective, simple and inexpensive biomarkers for osteosarcoma.

Our study has limitations. First, the study was retrospective, and it may provide significantly less evidence than a randomized controlled trial. Second, the antibodies used to detect D-dimer and fibrinogen levels were not identical, resulting in different optimal cut-off values. Third, the clinical factors, including ethnicity and age, in this study may also lead to bias.

Overall, this study demonstrated that preoperative elevated D-dimer and PF levels were associated with prognostic factors, including high clinical stage, metastasis and recurrence, that lead to poor OS and PFS rates in osteosarcoma. However, there was no significant correlation between the PF level and the prognosis of osteosarcoma. This study confirmed that the D-dimer level has an adverse effect on the clinicopathological features and metastasis of osteosarcoma. Thus, the D-dimer level can be used as an independent prognostic factor for OS and PFS in osteosarcoma patients. The D-dimer level may be a new candidate for osteosarcoma genotyping and a prognostic indicator of osteosarcoma patients.

## Data Availability Statement

The original contributions presented in the study are included in the article/**Supplementary Material**. Further inquiries can be directed to the corresponding authors.

## Ethics Statement

The studies involving human participants were reviewed and approved by The Ethics Committees of Wuwei City People’s Hospital. Written informed consent to participate in this study was provided by the participants’ legal guardian/next of kin.

## Author Contributions

All authors design the study, critical revision of the manuscript, and interpretation of data. YP and data acquisition and management and statistical analysis. YP, JW, and JSW drafting of the manuscript. SW study supervision. All authors contributed to the article and approved the submitted version.

## Conflict of Interest

The authors declare that the research was conducted in the absence of any commercial or financial relationships that could be construed as a potential conflict of interest.

## Publisher’s Note

All claims expressed in this article are solely those of the authors and do not necessarily represent those of their affiliated organizations, or those of the publisher, the editors and the reviewers. Any product that may be evaluated in this article, or claim that may be made by its manufacturer, is not guaranteed or endorsed by the publisher.
